# EphB6 overexpression and *Apc* mutation together promote colorectal cancer

**DOI:** 10.18632/oncotarget.9080

**Published:** 2016-04-28

**Authors:** Dan Xu, Liang Yuan, Xin Liu, Mingqi Li, Fubin Zhang, XinYue Gu, Dongwei Zhang, Youlin Yang, Binbin Cui, Jinxue Tong, Jin Zhou, Zhiwei Yu

**Affiliations:** ^1^ Department of Colorectal Surgery, The Affiliated Tumor Hospital of Harbin Medical University, Harbin, China; ^2^ Digestive System Department, The First Affiliated Hospital of Harbin Medical University, Harbin, China; ^3^ Division of Hematology, The First Affiliated Hospital of Harbin Medical University, Harbin, China; ^4^ Department of Surgery, The Second Affiliated of Harbin Medical University, Harbin, China

**Keywords:** EphB6, colorectal cancer, APC, biomarker, pathway analysis

## Abstract

The erythropoietin-producing hepatocyte (Eph) family tyrosine kinases play important roles in tumorigenesis and cancer aggression. In this study, we investigated the role of EphB6 in oncogenic transformation of colorectal epithelial cells *in vitro* and *in vivo*. EphB6 is upregulated in human colorectal cancer (CRC) tissues as compared to normal tissues, and its overexpression promotes proliferation, migration and invasion by IMCE colorectal adenoma cells, in which one *Apc* allele is mutated. EphB6 overexpression together with *Apc* mutation leads to the development of colorectal tumors *in vivo*. Expression microarrays using mRNAs and lncRNAs isolated from EphB6-overexpresssing IMCE and control cells revealed a large number of dysregulated genes involved in cancer-related functions and pathways. The present study is the first to demonstrate that EphB6 overexpression together with *Apc* gene mutations may enhance proliferation, invasion and metastasis by colorectal epithelial cells. Microarray data and pathway analysis of differentially expressed genes provided insight into possible EphB6-regulated mechanisms promoting tumorigenesis and cancer progression. EphB6 overexpression may represent a novel, effective biomarker predictive of cell proliferation, invasion and metastasis patterns in CRC tumors.

## INTRODUCTION

Colorectal cancer (CRC) is a heterogeneous disease, resulting from complex interactions between genetic and environmental factors. Risk factors for disease include old age, male sex, a low-fiber diet, smoking, drinking alcohol, diabetes, and genetic and environmental factors [1]. CRC is the third most common cancer and the fourth leading cause of cancer-related deaths worldwide [2]. In China, CRC ranks the fifth among cancer deaths, with a continually increasing incidence [3]. Despite surgical removal of the primary tumor, a significant proportion of CRC patients experience recurrence and may die within 5 years post-surgery [4]. Earlier diagnosis and discovery of recurrence after surgery are critical for effective treatment of CRC. Mechanisms underlying CRC development are currently unclear. Identification of novel diagnostic biomarkers sensitive and specific to CRC is urgently needed and would promote development of more effective therapeutics.

The erythropoietin-producing hepatocyte (Eph) receptors constitute the largest family of tyrosine kinase receptors in the human genome [5]. So far, the Eph receptor subfamily consists of 16 members, divided into two groups according to protein sequence, namely EphA1–A10 and EphB1–B6 [6]. Eph receptors mediate cell compartmentalization and directional cell migration during embryonic development [7, 8]. Given their abilities to control cell attachment and migration, Ephs are believed to play important roles in cancer invasiveness [9, 10]. Although the actions of some EphB receptors in cancer seem contradictory [11], EphB6 downregulation has been consistently correlated with enhanced aggressiveness and invasiveness in melanoma, neuroblastoma and non–small cell lung cancer [12–15]. However, the role of EphB6 in CRC has been poorly investigated. EphB6, an unusual Eph receptor without catalytic capacity due to alterations within its kinase domain, may act to suppress cancer aggression. However, the mechanisms underlying this action remain unclear.

The adenomatous polyposi coli (*APC*) tumor suppressor gene is homozygously inactivated in approximately 70% of all CRCs [16]. *APC* mutation is one of the earliest events in initiation and progression of CRC [17, 18]. Loss of *APC* function is observed throughout the development of intestinal carcinogenesis. In this study, we explored the synergism between EphB6 overexpression and *APC* mutations in the malignant transformation of a colonic adenoma cell line, IMCE (Immortomouse-Min Colonic Epithelial cells *Apc*^Min/+^), in which one copy of *Apc* is mutated.

Gene expression microarray data can be potentially applied to cancer research and treatment, such as tumor classification, study of tumor progression and metastasis, prediction of clinical outcomes and classification of drug resistance [19]. In this study, we show that EphB6 overexpression contributes to colorectal epithelial cell proliferation and CRC development in association with *APC* gene mutations. The role of EphB6 in the malignant transformation and evolution of colorectal epithelial cells was investigated through microarray, proteomics and bioinformatics analyses. Our data demonstrate the role of EphB6 in cancer-related signal transduction pathways and provide novel insight into mechanisms of CRC tumorigenesis.

## RESULTS

### EphB6 upregulation together with APC downregulation might contribute to tumorigenesis

**Figure 1:**
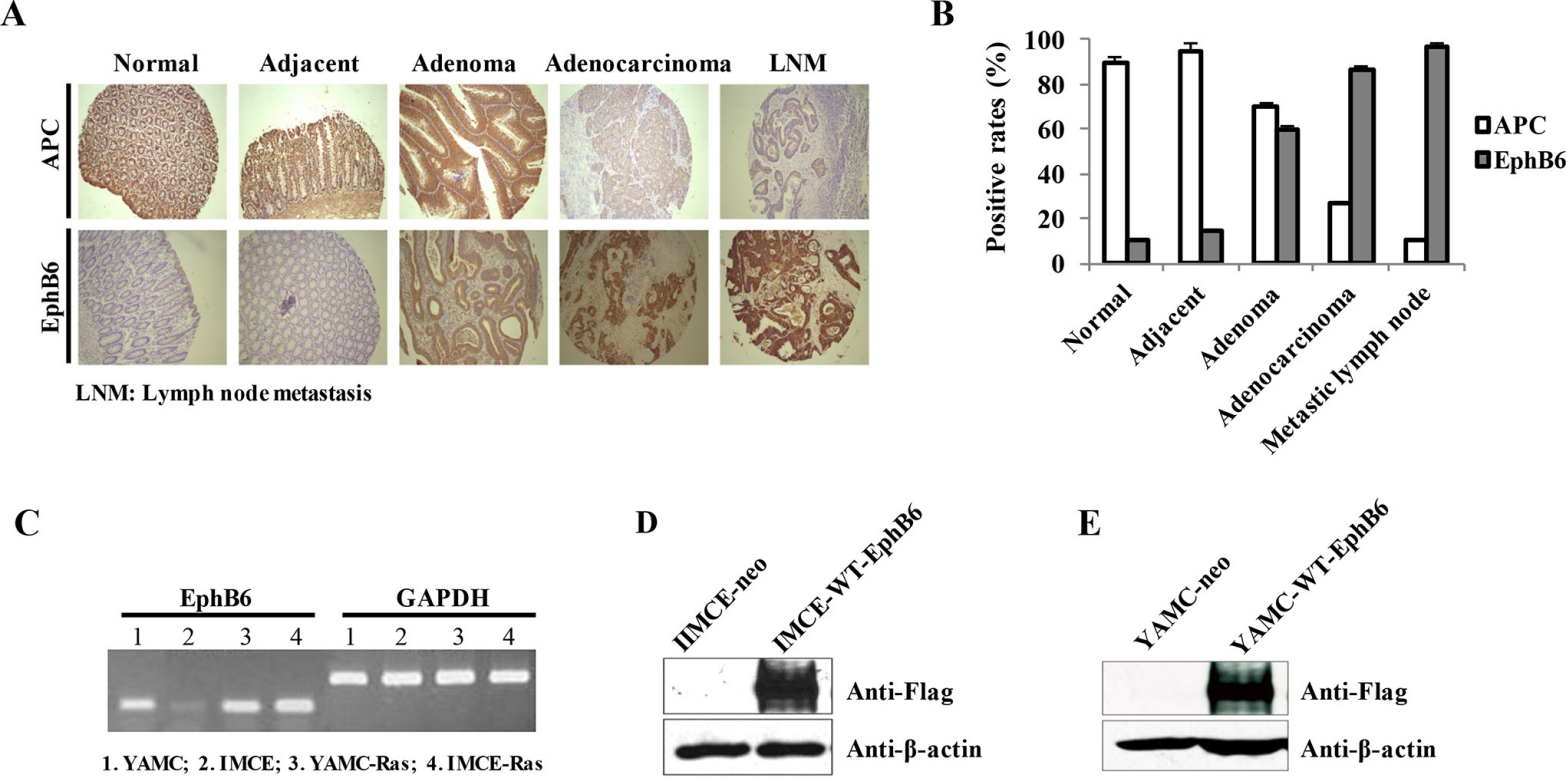
Establishment of EphB6 stably transfected IMCE and YAMC cell lines IHC was performed on the human tissue microarray using specific antibodies against APC and EphB6 (**A**). Rates of APC and EphB6 positivity in the human tissues for panel A (**B**). EphB6 mRNA was detected by RT-PCR in four rat colorectal epithelial cell lines including IMCE (*Apc*+/min) and YAMC (*Apc* +/+) (**C**). GAPDH was used as an internal control. Exogenous EphB6-Flag in the IMCE and YAMC stable cells was detected by western blot using Flag antibody (**D and E)**.

To investigate *in vivo* APC and EphB6 levels, we performed immunohistochemistry (IHC) on the human tissue microarray using specific antibodies. The microarray used in this study included 30 adenocarcinomas, 30 metastic lymph nodes, 10 adenomas and 30 normal tissue samples. APC was progressively downregulated and EphB6 was upregulated as disease progressed toward adenocarcinoma, but both were highly expressed in adenomas (Figure 1A and 1B, Tables 1 and 2). We hypothesized that the APC downregulation and EphB6 upregulation might be important for the malignant transformation of epithelial cells between these two CRC stages.

**Table 1:** APC and EphB6 levels in colorectal adenoma are correlated

		APC		*P*
		(+)	(−)	
EphB6	(+)	6	1	0.033
	(−)	0	3	

**Table 2:** APC and EphB6 levels in colorectal adenocarcinoma are not correlated

		APC		*P*
		(+)	(−)	
EphB6	(+)	8	18	0.55
	(−)	0	4	

### Establishment of EphB6 stably expressing IMCE and YAMC cell lines

RT-PCR results indicated that endogenous EphB6 expression in the IMCE cell line, which carries one mutated *Apc* allele, was very low (Figure 1C). We stably overexpressed EphB6 in IMCE cells by transfecting pcDNA3.1-EphB6-Flag and the empty vector into IMCE cells as well as a control colorectal epithelial cell line, YAMC, which contains wild-type *Apc*. Western blot data showed that exogenous EphB6-Flag was highly expressed in the IMCE and YAMC cells transfected with pcDNA3.1-EphB6-Flag, but not in the controls (Figure 1D and 1E).

### EphB6 overexpression promotes IMCE cell proliferation, migration and invasion

**Figure 2:**
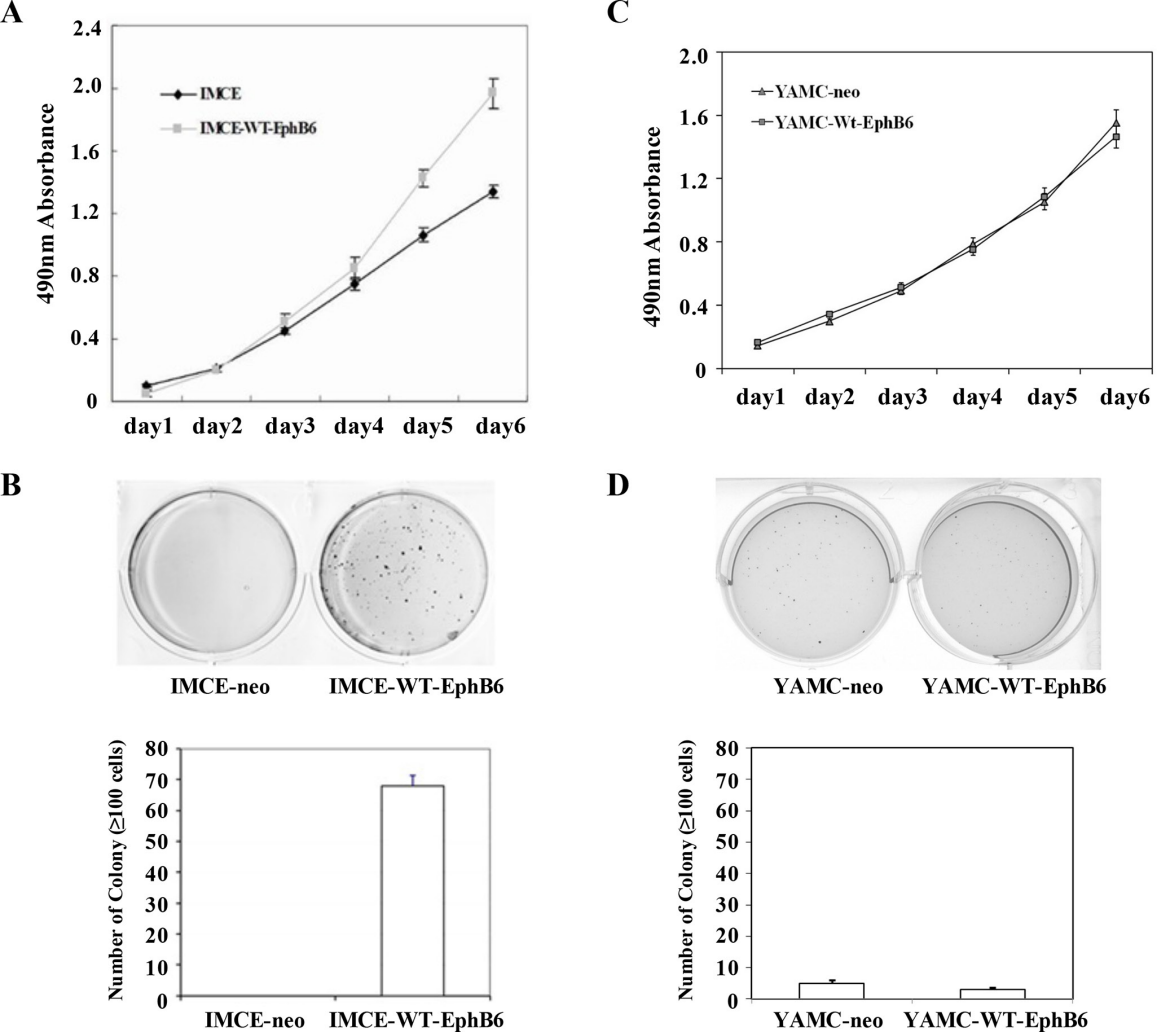
EphB6 overexpression enhances IMCE cell proliferation MTT (**A**) and colony forming (**B**) assays were performed using EphB6-Flag stably-expressing IMCE cells and empty vector-expressing controls, as well as YAMC cells (**C and D**).

**Figure 3:**
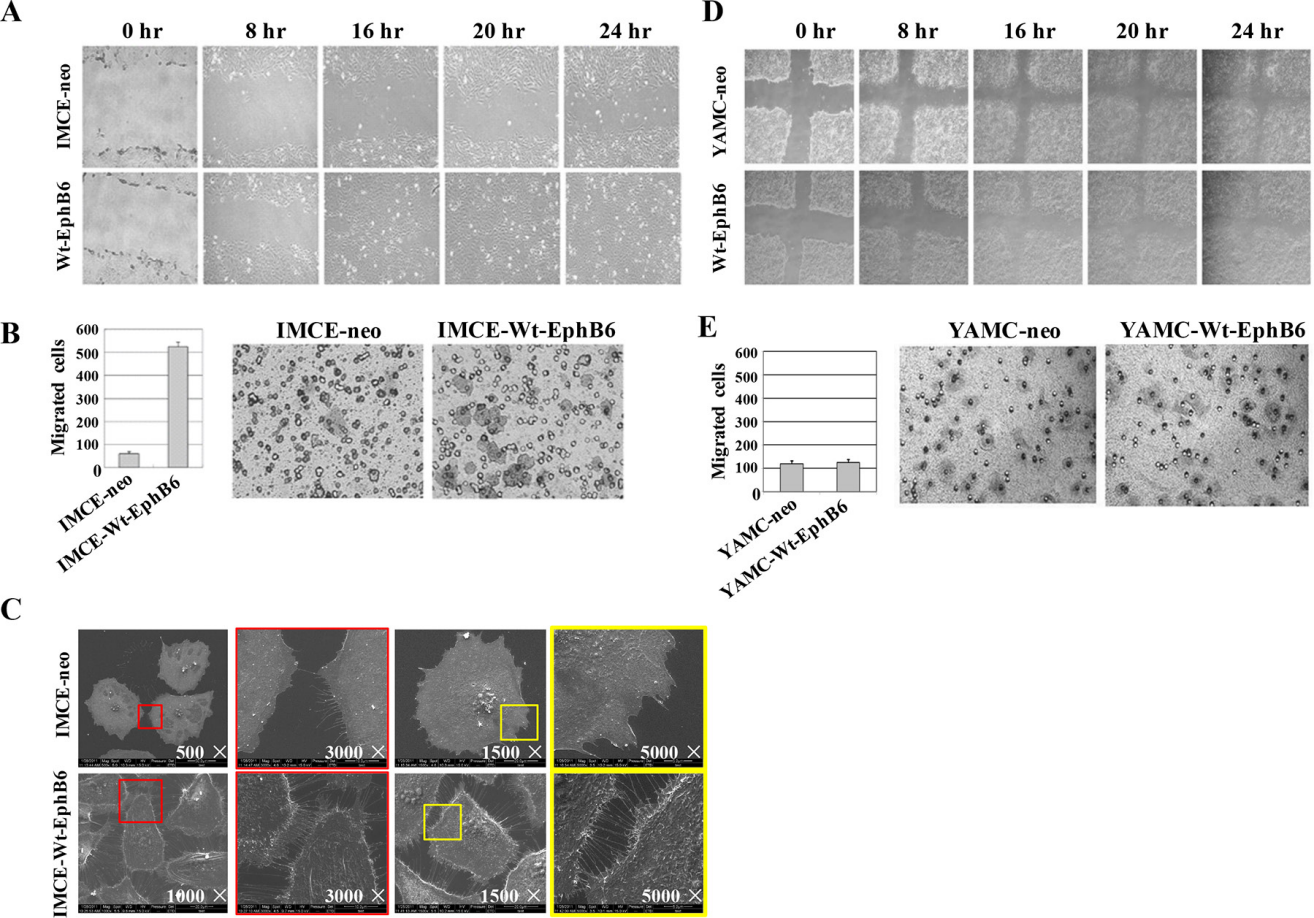
EphB6 overexpression promotes IMCE cell migration and invasion In wound-healing (**A**) and transwell (**B**) assays, EphB6 overexpression enhanced migration and invasion, respectively, in IMCE cells compared with controls. Cell morphology was altered in EphB6-overexpressing IMCE cells as was detected by scanning electron microscopy (SEM) (**C**) Wound-healing (**D**) and transwell (**E**) assays were performed for EphB6 over-expressing and control YAMC cells.

MTT and colony formation assay results showed that EphB6 overexpression promoted IMCE cell proliferation compared with controls (Figure 2A–2D). Wound-healing and transwell assays indicated that EphB6 overexpression promoted IMCE cell migration and invasion, respectively (Figure 3A and 3B). SEM micrographs revealed striking changes in IMCE cell morphology following EphB6 overexpression (Figure 3C). EphB6 overexpression did not enhance proliferation, migration or invasion in the control YAMC cells expressing wild-type *Apc* (Figure 3D and 3E).

### EphB6 overexpression in IMCE cells promotes tumorigenesis *in vivo*

**Figure 4:**
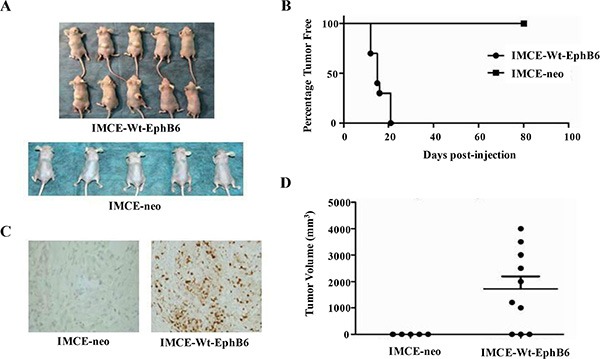
EphB6 overexpression enhances xenograft growth *in vivo* IMCE-neo or IMCE-WT-EphB6 cells were subcutaneously injected into athymic nude mice, which were sacrificed 80 days post injection (**A**) Percentage of tumor-free mice in the two groups (**B**) PCNA staining of xenografts (**C**) Tumor volume compared between the two groups (**D**).

Seven of ten mice injected with EphB6-overexpressing IMCE cells formed tumors by day 13, and all mice in this group had formed tumors by day 22, while mice injected with IMCE-neo control cells did not develop tumors (Figure 4A and 4B). Since aberrant cell proliferation is a major feature of the tumors, we then used PCNA (proliferating cell nuclear antigen), a commonly used cell proliferation marker, to stain the normal and tumor tissue samples from two mouse groups described above. Infiltrating lymphocytes in mice injected with IMCE-Wt-EphB6, but not IMCE-neo, cells showed positive PCNA staining (Figure 4C). In addition, tumor volume was significantly increased in mice injected with IMCE-Wt-EphB6 cells compared with the control (Figure 4D).

### Differential expression of mRNAs and lncRNAs in transfected IMCE cells

**Figure 5:**
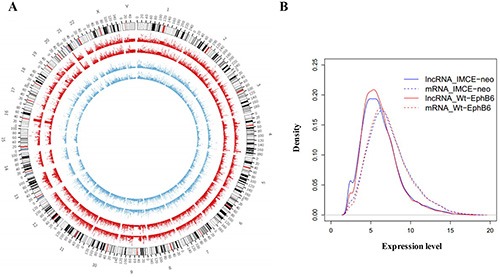
mRNA and lncRNA microarray expression profiles Circos v0.62 software was used to establish mRNA and lncRNA global expression profiles in IMCE-neo and IMCE-WT-EphB6 cells. Data are shown as a circular map (**A**) and probability density graph (**B**).

**Figure 6:**
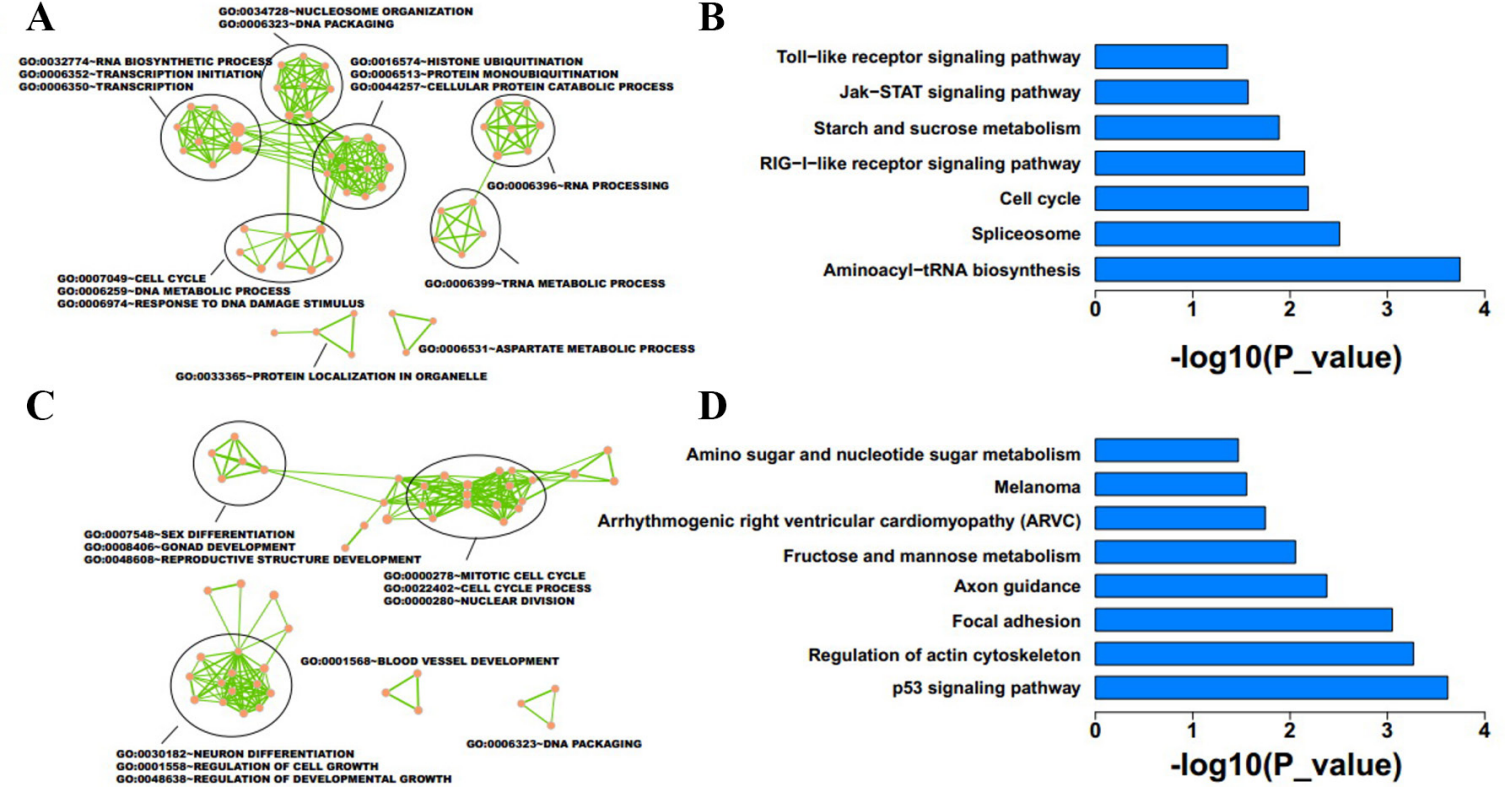
Function enrichment analysis was conducted for differentially expressed mRNAs in EphB6-overexpressing IMCE cells GO and KEGG enrichment analyses were performed using DAVID with *p* < 0.05. Results revealed the GO functions and KEGG pathways associated with upregulated (**A and B**) and downregulated (**C and D**) genes.

We performed microarray analyses to investigate global mRNA and lncRNA patterns in IMCE-neo and IMCE-Wt-EphB6 cells (Figure 5A and 5B). Although mRNA expression patterns between the two cell lines were very similar, a total of 1172 downregulated and 1425 upregulated genes were identified by differential expression analysis with a fold change ≥ 1.5. GO and KEGG analyses indicated that upregulated genes were mainly involved in the regulation of transcription, DNA packing and cell cycle progression (Figure 6A). These genes were enriched in seven pathways, including aminoacyl-tRNA biosynthesis, the spliceosome and the cell cycle (Figure 6B). In contrast, downregulated genes were involved in regulation of sex differentiation, the actin cytoskeleton, nuclear division, neuron differentiation, the p53 signaling pathway, focal adhesion signaling and other functions/pathways (Figure 6C and 6D).

**Figure 7:**
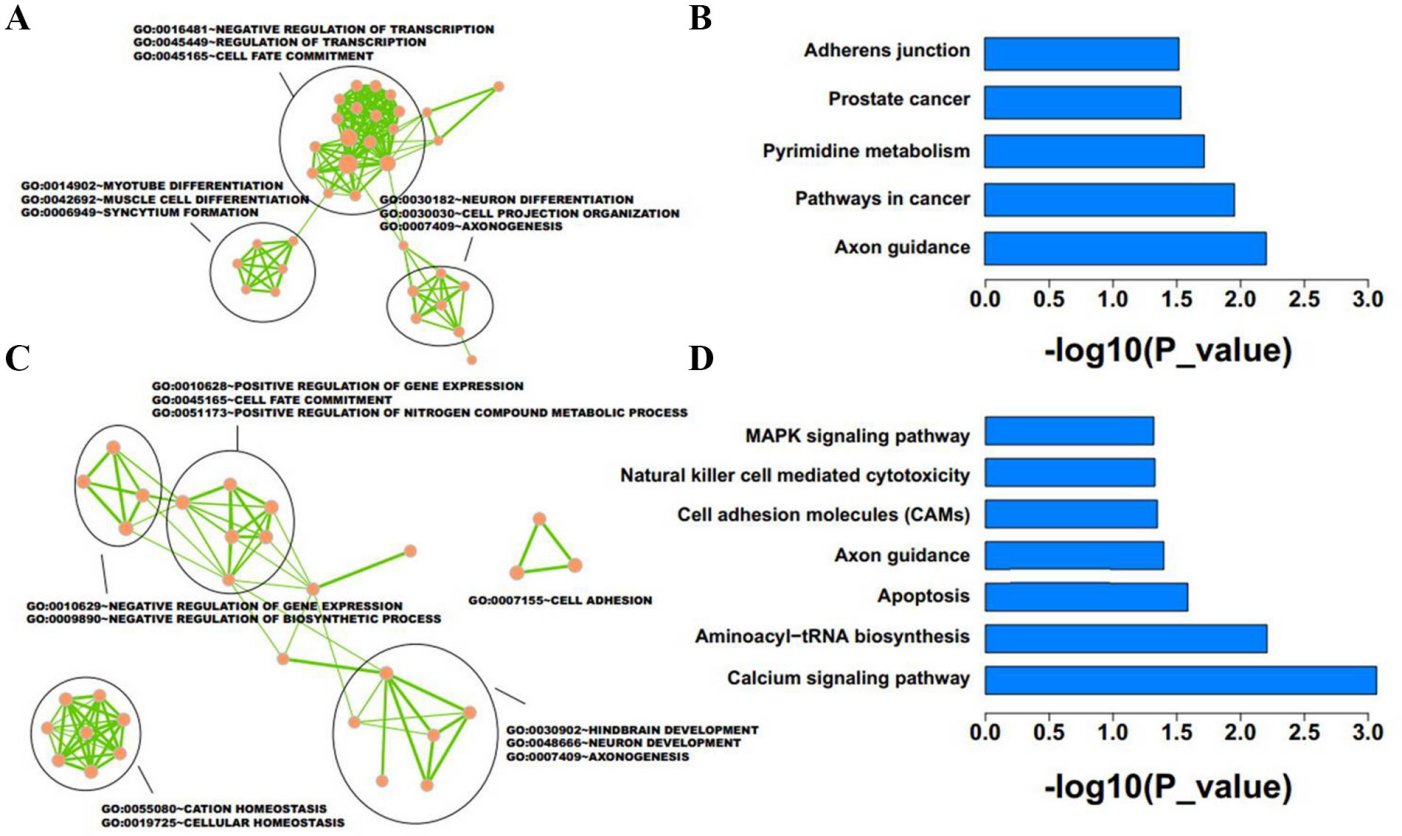
Function enrichment analysis was conducted for differentially expressed lncRNAs in EphB6-overexpressing IMCE cells Host or nearby genes of lncRNAs were selected for GO and KEGG enrichment analysis. Results revealed the GO functions and KEGG pathways associated with upregulated (**A and B**) and downregulated (**C and D**) lncRNA-neighboring genes.

A total of 1137 downregulated and 1714 upregulated lncRNAs were identified as differentially expressed between IMCE-neo and IMCE-Wt-EphB6 cells. Host or nearby genes for lncRNAs were identified and used for GO and KEGG enrichment analysis. Neighboring genes for upregulated lncRNAs were mainly involved in transcription regulation, myotube differentiation and muscle cell differentiation (Figure 7A), and these genes were enriched in five pathways, including axon guidance, cancer development, pyrimidine metabolism, and others (Figure 7B). However, downregulated lncRNAs were mainly important in positive regulation of gene expression and cell adhesion (Figure 7C), and enriched in seven pathways, including calcium signaling, aminoacyl-Trna biosynthesis, apoptosis, axon guidance and others (Figure 7D).

### Establishment of a subnetwork for common mRNAs and lncRNAs

**Figure 8:**
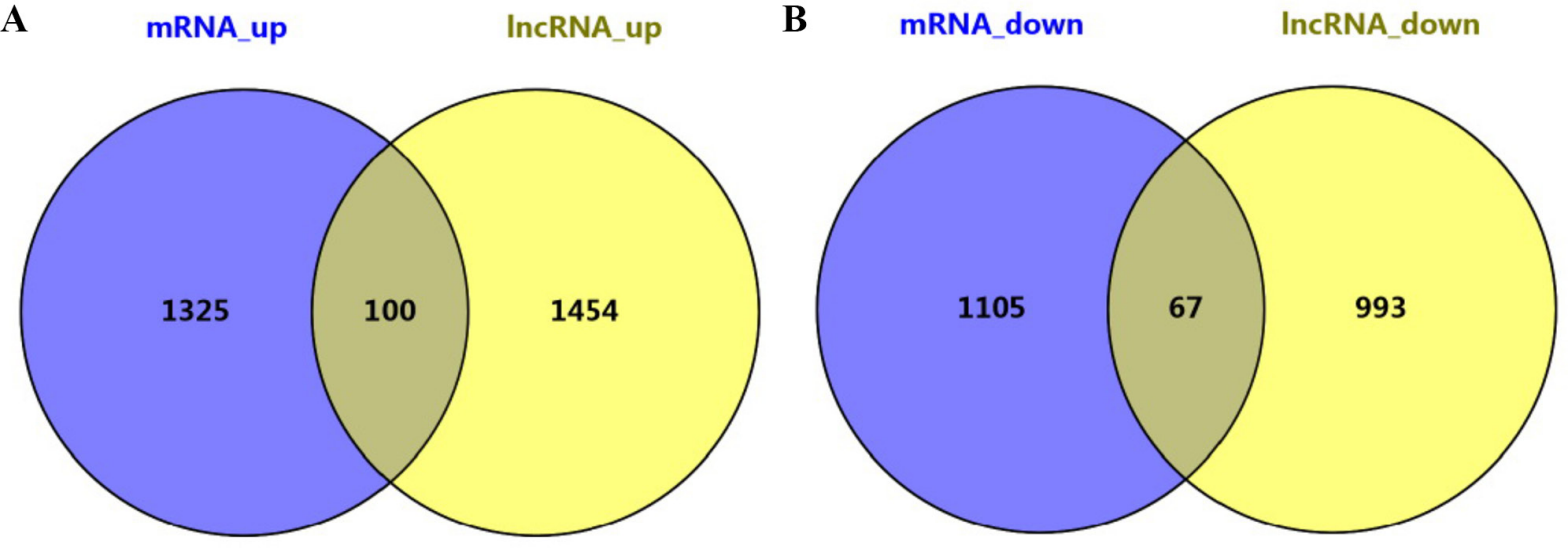
Differentially expressed lncRNAs and mRNAs in EphB6-overexpressing IMCE cells One-hundred genes were upregulated (**A**) and 67 were downregulated (**B**).

Among the mRNAs and genes near lncRNAs, 100 were upregulated while 67 were downregulated (Figure 8). Functional enrichment analysis indicated that these 167 genes were enriched in pathways such as RIG-I-like receptor signaling, the response to lipopolysaccharide and others (Table 3).

**Table 3:** Functional enrichment analysis of differentially expressed genes

Category	Term	Description	Count	Fisher
Kegg pathway	hsa04622	RIG-I-like receptor signaling pathway	3	0.016
Goterm bp fat	GO:0032496	response to lipopolysaccharide	4	0.0044
Goterm bp fat	GO:0045934	negative regulation of nucleobase, nucleoside, nucleotide and nucleic acid metabolic process	11	0.0048
Goterm bp fat	GO:0051172	negative regulation of nitrogen compound metabolic process	11	0.0053
Goterm bp fat	GO:0016481	negative regulation of transcription	10	0.0064
Goterm bp fat	GO:0002237	response to molecule of bacterial origin	4	0.0065

In recent years, the establishment of biological networks has become integral to the study of systems biology, as the protein-protein interaction (PPI) network is more accurate and straightforward in demonstrating gene relationships. The PPI network was integrated from eight PPI databases consisting of 80,980 edges and 13,361 nodes. The 167 genes that were consistently regulated at the mRNA and lncRNA levels were used as seed genes in the integrated network. In addition, the subnetwork included 1744 nodes and 1885 interactions, in which a small number of nodes were presented with high degrees (hub nodes) while the majority of the nodes were presented with low degrees. The hub genes in this subnetwork included GRB2, SMAD4, HDAC4, QKI, KPNA1, UBTF, ID2, MAD2L1, LSM1, MBD2, SNAPIN, FSCN1, ANXA1, ANAPC10, UBE2G2, SORBS2 and TARS (Table 4).

**Table 4:** Hub genes in the PPI network

Gene	lncRNA	Regulation	Degree
GRB2	RP11-16C1.3	up	527
SMAD4	LOC100287225	up	206
HDAC4	AC114788.2	down	114
QKI	RP11-300M24.1	up	42
KPNA1	KPNA1	up	41
UBTF	RP5-882C2.2	up	40
ID2	RP11-734K21.3	up	37
MAD2L1	RP11-96A1.5	up	32
LSM1	LSM1	up	30
MBD2	BC034434	up	30
SNAPIN	SNAPIN	down	29
FSCN1	ZNF815P	down	28
ANXA1	RP11-171A24.2	down	26
ANAPC10	ANAPC10	up	23
UBE2G2	AL773604.8	up	23
SORBS2	SORBS2	up	21
TARS	TARS	up	20

## DISCUSSION

As one of the largest tyrosine receptor families, Ephs are potential drug targets in the treatment of a variety of cancers [20]. However, the specific roles of the family members in tumor development remain poorly understood [21]. Ephrin-Bs/Ephs overexpression has been associated with tumorigenicity and poor prognosis in human osteosarcoma, endometrial cancer and melanomas [22]. EphB6 is a unique member in that it lacks kinase activity. Loss of EphB6 was associated with advanced tumor stage, cancer progression and tumor vasculature in several types of human cancers [23, 24]. In our study, we showed that endogenous EphB6 was upregulated in human colorectal adenomas and adenocarcinomas compared with normal tissues. We also showed that EphB6 overexpression promoted colorectal epithelial cell proliferation. *APC* alterations are among the first events associated with CPC development, and these alterations predispose individuals to additional gene alterations and tumorigenesis [25]. The present study is the first to demonstrate that EphB6 overexpression together with *APC* gene mutations may influence the behavior of colorectal epithelial cells, enhancing cell proliferation, invasion and metastasis.

In this study, we analyzed IMCE-neo and IMCE-Wt-EphB6 cell mRNA and lncRNA expression profiles via microarray using Circos software. Although our results showed similar expression patterns between the two cell lines, numerous striking differences were observed (Figure 5A and 5B) and differentially expressed genes were enriched in distinct functions and pathways (Figures 6 and 7). These data suggested that differentially expressed mRNAs and lncRNAs as a result of EphB6 overexpression might promote tumorigenicity. GO and KEGG analysis associated the differentially expressed genes with multiple potentially cancer-related functions and pathways, such as the cell cycle, p53 signaling, focal adhesion signaling, regulation of cell growth and others.

In conclusion, our results demonstrated that EphB6 overexpression may represent a novel, effective biomarker to predict cell proliferation, invasion and metastasis in CRC tumors. Furthermore, microarray data and pathway analysis of differentially expressed genes provided insight into possible EphB6-regulated mechanisms of tumorigenesis and cancer progression.

## MATERIALS AND METHODS

### Mice and cell cultures

Animal protocols were approved by the Institutional Animal Care and Use Committee (IACUC) of the Third Affiliated Hospital of Harbin Medical University. Nude mice, aged 5–6 weeks, were maintained in a pathogen-free environment in the experimental animal center of the Third Affiliated Hospital of Harbin Medical University. Rat colorectal YAMC, IMCE, YAMC-Ras and IMCE-Ras cell lines were kindly provided by Dr. Zhenfeng Zhang of Ireland Cancer Center, Case Western Reserve University (Cleveland, OH, USA). These cell lines were generated from colonic epithelia of F1 immorto-*APC*^min/+^ mouse hybrids. The immorto-mouse is an H-2Kb-tsA58 mouse expressing a heat-labile simian virus 40 large T antigen with an IFN-γ-inducible promoter. Cell lines were cultured in RPMI 1640 medium supplemented with 5% heat-inactivated fetal bovine serum (FBS), 5 U/ml of murine IFN-γ, 100 U/ml penicillin and streptomycin, 5 μg/ml insulin, 5 μg/ml transferrin, and 5 ng/ml selenium acid in a humidified incubator with 5% CO_2_ at 37°C.

### Reverse transcription-polymerase chain reaction (RT-PCR)

RNA was isolated from colorectal epithelial cell lines using Trizol reagent (Invitrogen, Carlsbad, CA, USA) and reverse transcribed into cDNA using Superscript First-Strand Synthesis System (Invitrogen) according to the manufacturers' instructions. PCR amplification was performed to detect EphB6 mRNA with specific primers. Forward primer: ACTGGATCCATGGTGTGTAGC CTATGGGTGCTGC; reverse primer: ACTGATATCTTAATCGACCTCCACTGA GCCCTGCTG. GAPDH was used as an internal control. The PCR protocol was as follows: 95°C, 2 min; 95°C for 30 s, 55°C for 45 s, and 72°C for 1 min, 30 cycles; 72°C, 15 min.

### Protein extraction and western blot

Whole cell lysates were prepared and quantified using standard protocols, separated via sodium dodecyl sulfate-polyacrylamide gel electrophoresis (SDS-PAGE) and blotted with a monoclonal Flag antibody (Cell Signaling Technology, Danvers, MA, USA). Membranes were then stripped and re-blotted with a monoclonal GAPDH antibody (Cell Signaling Technology).

### Establishment of IMCE and YAMC stable cell lines

Wt-EphB6 construct was kindly provided by Dr. Zhenfeng Zhang of Ireland Cancer Center, Case Western Reserve University (Cleveland, OH, USA). The EphB6 construct and empty vector plasmid DNA were individually transfected into the IMCE and YAMC cells using Lipofectamine 2000 (Invitrogen) according to the manufacturer's protocol. Twenty-four h post-transfection, cells were passaged at 1:5 and selected with G418 (Sigma, St Louis, MO, USA) for 2 weeks at gradually increased concentrations ranging from 200–800 μg/ml. Stably transfected clonal pools were harvested and expanded for subsequent experiments.

### Cell viability assay

Cell viability assay was performed using CellTiter 96^®^ AQ_ueous_ One Solution Cell Proliferation Assay kit (CellTiter96, Promega, Madison, WI, USA) according to the manufacturer's instructions. Briefly, stably transfected cells were seeded into 96-well plates and cultured up to 7 d. At each time point, 10 μL of MTT solution was added to the cells and incubated for 4 h, and then 150 μL of dimethyl sulfoxide (DMSO) was added to each well and mixed thoroughly. Optic density of each well was measured with a spectrophotometer (UV5100, Shanghai, China). Experiments were done in triplicate and repeated at least three times.

### Clonogenicity assay

One thousand cells were initially seeded in a 6-well plate using a 2-layer soft agar system as previously described [26]. After three-weeks at 37°C in 5% CO_2_, cells were fixed with methanol and stained with 1% crystal violet (dissolved in 20% methanol) for 20 min. Colonies were counted under an inverted microscope. All experiments were done in triplicate and repeated at least three times.

### Wound healing assay

A total of 2 × 10^5^ cells were seeded into each well of a 12-well plate with 500 μL of DMEM (containing 10% FCS). At 100% cell confluency, a scraped line (wound) was created using a pipette tip and scratch widths were marked. Cells were washed with iced-cold PBS and further cultured in 500 μL of DMEM medium at 37°C for 24 h. Wound closure was imaged under an inverted microscope.

### Transwell migration and invasion assay

Transwell filter inserts (8-μm pore size) for 24-well plates were purchased from Costar (Cambridge, MA, USA). In brief, 5 × 10^4^ cells in 500 μL of DMEM (containing 1% FCS) were seeded in the upper chamber and 500 μL of DMEM (containing 10% FCS) was added to the lower chamber. Cells were incubated at 37°C for 24–36 h. Cells remaining on the surface of the upper chamber were removed by a cotton swab while cells migrated into the bottom of the filter were fixed with 4% formaldehyde and stained with 0.5% crystal violet. Cells were counted in five photographed fields.

In the invasion assay, transwell filters were precoated with Matrigel and the assay was performed following the migration assay protocol. Both cell migration and invasion assays were performed in triplicate and repeated three times.

### Scanning electron microscope

Cells were fixed in 2.5% glutaraldehyde for 24 h, dehydrated with ethanol at graded concentrations and dried in CO_2_ at the critical point. Dried samples were mounted on aluminum, sputter-coated with gold and examined under a scanning electron microscope.

### *In vivo* tumorigenic assays

Xenograft assays were performed to investigate the tumorigenic potential of modified IMCE cells in mice. Mice were divided into two groups. Group A was injected subcutaneously with 8 × 10^8^ IMCE-neo cells, and group B was injected with the same number of IMCE-Wt-EphB6 cells. Tumor volume (V, cm^3^) was evaluated after 4 weeks based on tumor length (l), width (w) and height (h) (V = l × w × h × 0.5236).

### Microarray assay and data processing

Microarray assays were performed to establish differential gene expression profiles in IMCE-neo and IMCE-WT-EphB6 cells. The mRNA microarray contained 23,420 probes corresponding to 17, 298 genes. Probes matched to multiple genes were eliminated. Probe expression values represented gene expression levels and values generated from multiple probe sets were averaged. The lncRNA microarray included 34,735 probes. If the lncRNA matched to a single probe, its expression level was same as that of the probe; if the lncRNAs matched to several probes, its expression level was the same as that of the longest probe. Expression values for 16,746 lncRNAs in total were presented.

### Identification of differentially expressed genes and lncRNAs

Differentially expressed genes and lncRNAs between IMCE-neo and IMCE-WT-EphB6 cells were those with fold changes ≥ 1.5. Genes close to the lncRNAs were filtered out using the human refSeq reference genome (hg19). Genes located most closely to the region 500 kb upstream from the transcription start site (TSS) were considered to be potentially affected by lncRNAs.

### Functional analysis of differentially expressed genes and lncRNAs

Gene Ontology (GO) and Kyoto Encyclopedia of Genes and Genomes (KEGG) functional enrichment analysis was performed using DAVID Functional Annotation Bioinformatics Microarray Analysis (http://david.abcc.ncifcrf.gov/home.jsp) for differentially expressed genes, and the hosts or nearby genes of differentially expressed lncRNAs, respectively. Fisher's exact test was applied to determine statistical significance. The EnrichmentMap plugin in Cytoscape was adopted for GO_BP term enrichment analysis.

### Construction of protein-protein interaction subnetworks for lncRNAs and mRNAs

An integrated protein-protein interaction (PPI) network was established based on the following databases: Biomolecular Interaction Network Database (BIND), the Biological General Repository for Interaction Data sets (BioGRID), the Database of Interacting Proteins (DIP), the Human Protein Reference Database (HPRD), IntAct, the Molecular INTeraction database (MINT), the mammalian PPI database of the Munich Information Center on Protein Sequences (MIPS), PDZBase (a PPI database for PDZ-domains) and Reactome. The common mRNAs and the nearby genes for lncRNAs were set as seed genes. The subnetwork was derived from the seed genes, nearby lncRNAs and the genes connected to the seed genes in the PPI network. The network was constructed using Cytoscape (http://www.cytoscape.org/), open-source software used to establish biological networks.

### Statistical analysis

Statistical analysis was performed using SAS software (version 9.2). The paired *t* test was used to compare differences between IMCE-neo and IMCE-WT-EphB6 cell migration, and the χ^2^ test was used to analyze data from cell proliferation, clonogenicity and tumorigenic assays *in vivo*. *P* < 0.05 was considered statistically significant.
